# Randomized Double-blind Placebo-controlled Proof-of-concept Trial of Resveratrol for Outpatient Treatment of Mild Coronavirus Disease (COVID-19)

**DOI:** 10.21203/rs.3.rs-861831/v1

**Published:** 2021-09-13

**Authors:** Marvin R. McCreary, Patrick M. Schnell, Dale A. Rhoda

**Affiliations:** Mount Carmel Health Systems; The Ohio State University; Biostat Global Consulting

**Keywords:** SARS-CoV-2, COVID-19, coronavirus, resveratrol, polyphenol, stilbene, phytoalexin, vitamin D3, cholecalciferol, supplement, micronutrient, clinical trial, PRO-CTCAE

## Abstract

Resveratrol is a polyphenol that has been well studied and has demonstrated anti-viral and anti-inflammatory properties that might mitigate the effects of COVID-19. Outpatients (N=105) were recruited from central Ohio in late 2020. Participants were randomly assigned to receive placebo or resveratrol. Both groups received a single dose of Vitamin D3 which was used as an adjunct. The primary outcome measure was hospitalization within 21 days of symptom onset; secondary measures were ER visits, incidence of pneumonia and pulmonary embolism. Five patients chose not to participate after randomization. Twenty-one day outcome was determined of all one hundred participants (mean [SD] age 55.6 [8.8] years; 61% female) (or their surrogates). There were no clinically significant adverse events attributed to resveratrol. Outpatients in this phase 2 study treated with resveratrol had a lower incidence compared to placebo of: hospitalization (2% vs. 6%, RR 0.33, 95% CI 0.04-3.10), COVID-related ER visits (8% vs. 14%, RR 0.57, 95% CI 0.18-1.83), and pneumonia (8% vs. 16%, RR 0.5, 95% CI 0.16-1.55). One patient (2%) in each group developed pulmonary embolism (RR 1.00, 95% CI: 0.06-15.55). This underpowered study was limited by small sample size and low incidence of primary adverse events. A larger trial could determine efficacy.

**TRIAL REGISTRATIONS:**

ClinicalTrials.govNCT04400890 26/05/2020; FDA IND #150033 05/05/2020

## Introduction

### Study Rationale

**Figure 1:**
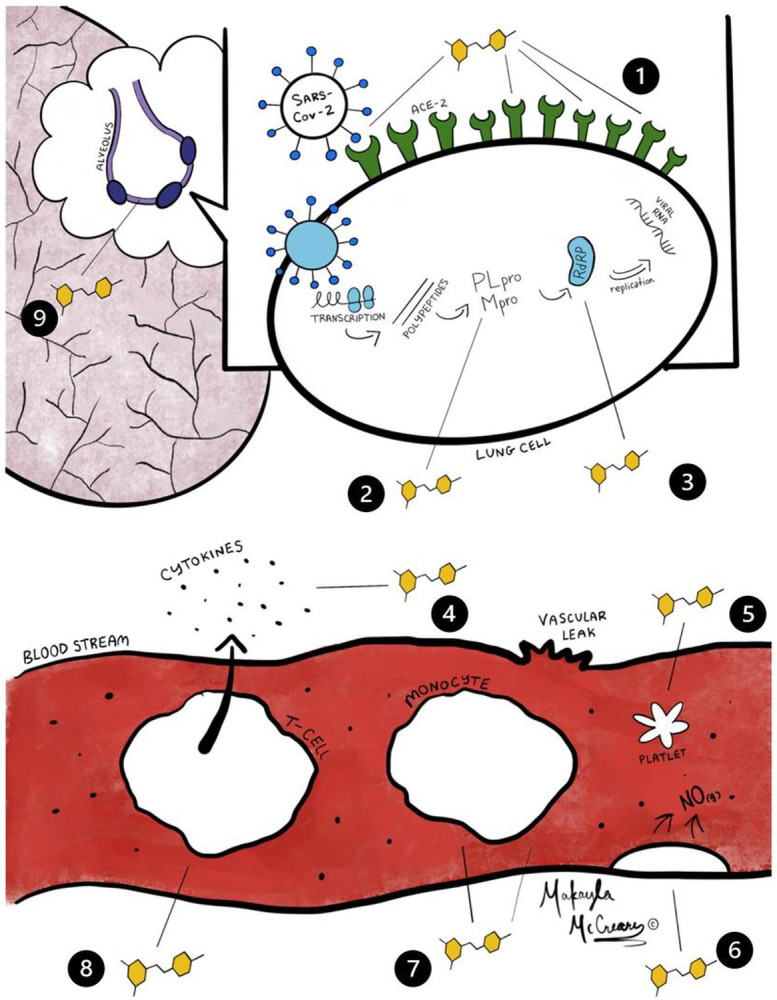
Summary of potential resveratrol effects on virus and host (See Figure key) Figure 1 Key 1. Inhibits Spike protein to ACE2 binding 4,5 2. Inhibits transcription of viral proteases (Mpro and PLpro) 6–9 3. Inhibits RNA-dependent RNA polymerase 10 4. Inhibits proinflammatory cytokines 11–13 5. Inhibits platelet aggregation 14,15 6. Activates endothelial Nitric Oxide (antiviral and vasoprotective) 16–18 7. Inhibits proinflammatory NF-kB 19 8. Inhibits proinflammatory Th-17 T-cells 20 9. Stimulates the production of glutathione in lung epithelium 21–23

Resveratrol (RV) is a polyphenolic phytoalexin produced by certain plants in response to injury or infection. RV has been associated with a variety of positive health effects in areas of inflammation, cardiovascular diseases, cognitive disease, cancer, diabetes, and infectious disease (including viral diseases) ^1,2^. RV is readily available commercially as a dietary supplement produced from plant extracts or by genetically engineered yeast ^3^. COVID-19 is the disease caused by a novel coronavirus (SARS-CoV-2) that can result in life threatening complications, including lung injury. Outpatient treatment options for COVID are limited. Multiple lines of preclinical data suggest that RV could be effective against coronavirus disease 2019 (Figure 1).

### Background

SARS-CoV-2 is characterized by surface spike proteins that bind to the Angiotensin-Converting Enzyme 2 (ACE2) of the respiratory tract. After entry into the cell, a variety of processes occur, including the down regulation of ACE2, subsequent destruction of the pneumocyte, the release of inflammatory mediators, and the subsequent release of cytokines (IL1, IL6, TNF-α) and reactive oxygen species ^24,25^. A “cytokine storm” results in further damage to the alveoli and the development of Acute Respiratory Distress Syndrome (ARDS) ^25^. Resveratrol’s multimodal antiviral, anti-inflammatory, and antioxidant properties as well as its ability to upregulate ACE2 receptors could be helpful in reducing the clinical effects of COVID.

### ACE2 Upregulation

In addition to ACE2 being a binding site for coronavirus (CoV), it is also associated with protective effects in SARS induced lung injury ^26,27^. ACE2 may attenuate vascular permeability, inflammatory cell infiltration, pulmonary edema, hyaline membrane formation, and prevent acute lung injury ^28^. Resveratrol has been shown to upregulate ACE2 ^29^. A deficiency of ACE2 caused by SARS is associated with lung injury ^28^. The upregulation of ACE2 by resveratrol might provide protective effects in COVID-19 ^28,30-32^.

### Anti-viral Effects

RV has demonstrated antiviral effects in a variety of animal and human disease ^2^. Specific to CoV, in vitro studies demonstrate that RV inhibits MERS-CoV infection by decreasing nucleocapsid protein resulting in reduced viral production and increased cell survival ^33^. Starting at the first steps in the infection in silico modeling suggests that RV would interfere with the binding of CoV spike protein to the ACE2 receptor (Figure 1) ^4,5^. In silico analysis also suggests that RV would inhibit COVID-19 RNA Dependent Polymerase and Papain-like Protease (PLpro) (Figure 1) which could explain the inhibition of nucleocapsid protein described by Lin et al 2017 ^6,10,33^. in silico analysis also demonstrates potential inhibition of the coronavirus main proteinase (Mpro) which would be additional mechanism of inhibiting viral replication^7^.

### Anti-inflammatory effects

COVID-19 is associated with the potential for excessive inflammation. Coronavirus has been shown to activate Toll-Like Receptor 4, increase pro-inflammatory cytokines IL-1, IL-6, CCL5 (RANTES) and TNF-α leading to an unbalanced inflammatory response and damaging inflammation ^34-37^. in contrast, RV has been shown to reduce inflammation via a variety of mechanisms (Figure 1) ^11-13,38^. RV has been demonstrated to inhibit TLR4 activation, decreasing the release of inflammatory cytokines in the macrophages of patients with COPD, and inhibit the proinflammatory transcription factor NF-κB ^14,19,39^. RV has also demonstrated inhibition of pro-inflammatory Th17 helper T-cells (Figure 1)^20^. Inhibition of NF-κB has been shown to increase survival in a mouse model of SARS-COV1 ^40^.

The anti-inflammatory effects of RV might be beneficial in mitigating the cytokine storm that is associated with ARDS and high mortality of COVID-19 ^25^. A mouse model of cytokine storm showed a 100% mortality reduced to 0% in RV treated mice with minimal lung injury in the treated group ^41^. RV has demonstrated protective effects in LPS induced lung injury, a mouse model of ARDS ^42,43^. The proposed mechanism is RV’s inhibition of NLRP3 inflammasomes ^42^. Inhibition of NLRP3 inflammasomes in another proposed therapeutic target in COVID-19 ^44^.

### Antioxidant Effects

Depletion of the endogenous antioxidant glutathione has been attributed to poor outcomes and death in patient with COVID-19 (Figure 1) ^21^. The use of antioxidants has been proposed in the treatment of COVID-19 ^45^. RV’s antioxidant properties as well as its ability to induce glutathione synthesis might provide additional outcome benefits ^22^.

### Animal Models of Viral Infections

As the above discussion regarding RV’s effects are largely based on in vitro models of disease, there is always a concern regarding whether in vitro models will translate into in vivo efficacy. Multiple animal studies have shown that RV does improve outcomes in animal models of viral infections. A porcine model of pseudorabies virus, a respiratory illness, shows that piglets inoculated with the virus had no mortality compared to a 40% mortality in the untreated group. Specifically, that study showed alveolar destruction in the untreated group vs mild lung injury in the treated group. The proposed mechanism is inhibition of IκB kinase by RV ^46^. It is notable that a drug prediction analysis of SARS-CoV-2 suggests that IκB kinase inhibition is a potential target for COVID-19 ^47^. Similarly, a murine model of H1N1 influenza showed a 60% survival rate in RV treated mice compared to 20% in placebo treated mice ^48^. In Respiratory Syncytial Virus (RSV) infected mice, RV treated mice showed significantly less lung damage compared to untreated mice ^49^.

### Vitamin D3

Vitamin D3 was included in the treatment protocol as an adjunct to RV based upon prior research showing that it has synergistic anti-inflammatory effects, inhibiting IL-6 and TNF-α ^11^. Both treatment arms received a single 100,000 IU dose of D3 to quickly assure adequate serum concentrations of D3, as well as to potentially remove vitamin D3 deficiency as a confounding variable, noting that multiple publications raised concerns that vitamin D3 deficiency might be associated with worse outcomes in COVID-19 ^50,51^.

## Materials And Methods

### Study Design

#### Overview

This study was a phase 2, double-blind, randomized, placebo-controlled trial to evaluate the safety and explore the efficacy of RV plus vitamin D3 based on the hypothesis that RV with the adjunct vitamin D3 can reduce hospitalization and morbidity in patients with COVID-19. The study was approved by the U.S. Food and Drug Administration as an investigational new drug trial (**FDA IND #150033 05/05/2020; ClinicalTrials.gov NCT04400890 26/05/2020)**, and the intuitional review board of Mount Carmel Health Systems in Columbus, Ohio, USA. All patients were provided informed consent and screening remotely via phone interview, educated via online animated presentation, and e-consented via REDCap electronic data capture tools hosted at the Ohio State University Medical Center and incorporated questions from the REDCap Shared Library ^52,53^.

Patients were recruited primarily from the Mount Carmel Health System testing centers by way of “cold calls” to patients 45 and older who tested positive for COVID-19. A few patients were recruited in response to research advertisements in the central Ohio area (social media, radio, and yard sign advertising), as well as physician referrals. Due to pandemic related safety concerns, the patients remained in quarantine within their home with all trial contact via phone, email, and web (REDCap), with contactless delivery of study packets via courier/mail. Packets were delivered within 7 days from the onset of symptoms, typically < 24 hours after consent signed.

Due to reports of patients self-medicating with investigational drugs (e.g., hydroxychloroquine) in the setting of COVID-19, the specific nature of the trial substance was concealed from subjects until after the study was complete. Patients were informed that they were receiving a “commercially available dietary supplements”, but the use of RV was not disclosed. The use of Vitamin D3 was open-label for both groups.

Patients were provided with a study packet containing identically prepared capsules containing a 15-day supply of either resveratrol or placebo, a one-time dose of vitamin D3, a thermometer, a pulse oximeter, and an instruction booklet with dosing log.

Data was collected via REDCap surveys on days 1-15, 21, 30, and day 60 with adverse symptoms assessed using selected PRO-CTCAE questions ^54^. All patients were given daily online reminders of when to seek medical care based upon CDC recommendations. Primary and secondary outcome measures (including hospitalization, ER visits, history of chest imaging, and pneumonia) were assessed by phone interviews after 21 days from randomization. All radiology reports were reviewed by the principal investigator.

#### Sample Size Determination

The maximum total number of randomized subjects was capped at 200 by FDA request. Power analyses were conducted for the primary outcome measure (hospitalization) assuming multiple placebo arm hospitalization rates and effect sizes, as well as for secondary outcome measures.

At the time the protocol was developed, the rate of hospitalization among confirmed cases of COVID-19 ranged between 21% in the 45-54 age bracket, up to 31% for patient’s >85 ^55^ . A planned sample size of 190 subjects with complete observations yielded 80% power at the 5% two-sided significance level to detect a difference in the primary endpoint (hospitalization) rate of 10% in the resveratrol arm versus 25% in the placebo arm.

An interim analyses was completed by an independent data and safety monitoring board. The analayis used the Hwang-Shih-DeCani alpha spending function with parameter gamma = −4 (O’Brien-Fleming–like) for the upper (superiority) bound under the null hypothesis with total one-sided Type I error 2.5%, and for the lower (safety or futility) bound under the alternative hypothesis with total Type II error 20% (80% power). Under the assumption of a binding futility bound and a placebo arm hospitalization rate of 25%, the probability of declaring futility at the interim analysis is 3% if the resveratrol arm hospitalization rate is 10% (alternative hypothesis), 55% if the resveratrol arm hospitalization rate is 25% (null hypothesis), and 75% if the resveratrol arm hospitalization rate is 30%. The R package gsDesign was used to determine stopping boundaries.

#### Participants

Due to low risk of hospitalization (the primary outcome measure), patients younger than 45 were excluded ^55^. Patients were eligible for enrollment if they tested positive for SARS-CoV-2 and had symptoms for less than 7 days by the expected delivery date of study packet. Exclusion criteria included cognitive impairment that would prevent the patient from cooperating with study procedures; asymptomatic patients; known history of cirrhosis, hepatic impairment, or Hepatitis C; known of history of renal impairment as measured by an eGFR of < 60 mL/min; patients receiving chemotherapy or who are on chronic immunosuppressants; allergy to grapes or rice; co-morbidities with a high likelihood of hospitalization within 30 days; currently pregnant; hospitalization; patients taking immunosuppressants and drug interactions in medications with a narrow therapeutic index. Patients on “statins” and PDE-5 inhibitors were instructed to withhold while on the study treatment.

It is notable that the renal disfunction exclusion was an FDA requirement. Prior research has explored possible benefits of RV for patients with chronic kidney disease ^56^. Furthermore, increased plasma levels of RV that might be attained in the setting of kidney disease might be beneficial.

#### Randomization

The random allocation list was blocked and stratified by a third-party group. Randomization used balanced blocks of size 2 or 4, selected randomly for each block. Randomization schedules were generated and rejected until the randomization schedule was balanced at 100, 200, and 210 subjects to align with the planned interim and final analyses, and in case of a 10-subject overrun. During the trial, only the third-party group and Data Safety Monitoring Board (DSMB) had access to the randomization list. The study personnel created identical-looking packets with identical-appearing study agents containing a 15-day dosing regimen according to the random allocation list. Study personnel were blinded to the contents of the distributed packets, with bottles only differentiated by a tamper-resistant serial number label applied by the third-party group which corresponded to the randomization list.

#### Blinding

A disinterested third party (Capital University, Department of Mathematics, Columbus, Ohio) was hired to assign tamper resistant serial number stickers as either RV or placebo based upon the output of randomization script from the R statistical software ^57^. The third party, using a two-person team to provided validation, assigned serial numbers to appropriate manufacturer sealed RV or placebo bottles. The prepared bottles were returned to the research team such that the bottle could only be differentiated by the serial numbers. The randomization table of the serial number labels was kept only by the third party and the Data Safety Monitoring Board until the completion of the study.

#### Intervention

Patients received identically appearing bottles containing 60 identically appearing capsules of either >98% pure trans-resveratrol (from Japanese Knotweed Root, Polygonum cuspidatum extract) (500mg per capsule) or placebo (brown rice flour) (both prepared and bottled by Vita-Age, Vancouver, BC) with instructions to take 2 capsules 4 times per day for at least 7 days, and up to 15 days if COVID symptoms persisted. Dosing was determined based upon publish IC50 of resveratrol against MERS-COV and previously published pharmacokinetic literature of resveratrol plus its metabolites. (See the study protocol PDF at www.clinicaltrials.gov/ct2/show/NCT04400890 for detailed dose justification and products certificates of analysis.)

#### Participant Monitoring and Follow-up

Starting on day 1, and continuing daily for 15 days, subjects were contacted via automated e-mail. Messages includes a reminder to take their study medication as scheduled and complete the daily surveys. Subjects were asked to complete a short questionnaire covering: 1) symptoms they had that day that could be related to COVID-19 (e.g., fever, cough, dyspnea), their frequency and severity; 2) report any other related or non-related medical events; 3) any medications they have taken to relieve symptoms, or other new medications they have not previously reported to study personnel; and 4) any visits they have made to healthcare providers, outpatient centers or hospitals, and details regarding those visits. Subjects received reminders when to seek care if they experience symptoms that are worsening or that are concerning to them.

Participants were sent a PRO-CTCAE questions on days 1, 8, 15, 21, 30, and 60 to monitor adverse events. All subjects provided a surrogate/secondary contact (spouse/family member/friend) in order to determine the subject’s status if the subject could not be reached. All patients or their secondary contact were interviewed for follow up after 21 days from randomization (no participants were lost to follow up for their post-21 day follow up brief interview).

#### Endpoints

Hospitalizations were determined based on query of subject or the subject’s secondary contact, and the patient’s medical records. Additional outcomes include assessing number of days with fever, and to assess symptoms, including dyspnea and fatigue. Questionnaires to assess symptoms and adverse events were based on the PRO-CTCAE (Supplemental Tables S-2b, and S-3b) ^54^.

### Statistical Analysis

#### Data management

Anonymized data were extracted from REDCap and processed into a dataset with one row per participant. Self-reported symptom and adverse event data were retained for every patient contact over the 21 days following randomization. Data were analyzed using Stata version 17 ^58^.

#### Primary analysis (including missing data)

The primary analysis is a comparison of the proportion of persons in the two groups who were hospitalized within 21 days of symptom onset. The comparison was evaluated using Fisher’s exact test, considering the difference to be statistically significant if the two-sided p-value is smaller than 0.05. The analysis uses the intent-to-treat method where all participants are analyzed as part of their randomization group, regardless of whether and when they withdrew from the study and regardless of whether or how well they complied with the study protocol. Missing outcome data were subject to tipping point sensitivity analysis to understand what distribution of missingness, if any, would change the conclusion reached using complete case analysis ^59^.

#### Secondary analyses (including missing data)

Secondary outcomes were analyzed using Fisher’s exact test, also, with no adjustment for multiple comparisons. Those outcomes were also subject to tipping point analysis of missing outcome data.

#### Sub-group analyses

The primary and secondary endpoints were analyzed among planned sizable sub-groups using Fisher’s exact test with no adjustment for multiple comparisons.

#### Adverse events

PRO-CTCAE questions vary in format to either recording the presence or absence of symptoms, or to grading the frequency, severity, and interference in activities of daily living of symptoms. Severity is graded as 0 = None, 1 = Mild, 2 = Moderate, 3 = Severe, 4 = Very Severe. Frequency is graded as 0 = Never, 1 = Rarely, 2 = Occasionally, 3 = Frequently, 4 = Almost constantly. Interference is graded as 1 = Not at all, 2 = A little bit, 3 = Somewhat, 4 = Quite a bit, 5 = Very much. Presence is graded as 0 = No, 1 = Yes ^54^.

Participants were asked about symptoms a) at enrollment (current symptoms), b) in a daily diary during 15 days of treatment (symptoms today), and c) on days 1, 8, 15, and 21 of the study (over the past 7 days). For questions about presence of a symptom, prevalence was compared using Fisher’s exact test. For questions about severity, frequency, or interference with activities of daily living (ADL), the proportion who answered 1+ and the proportion who answered 3+ were compared using Fisher’s exact test. Responses at enrollment or on day 1 of the study were used to characterize differences between study groups at baseline. Responses on days 2-21 were used to characterize differences in effects of placebo vs. resveratrol.

## Results

### Study participants

**Figure 2:**
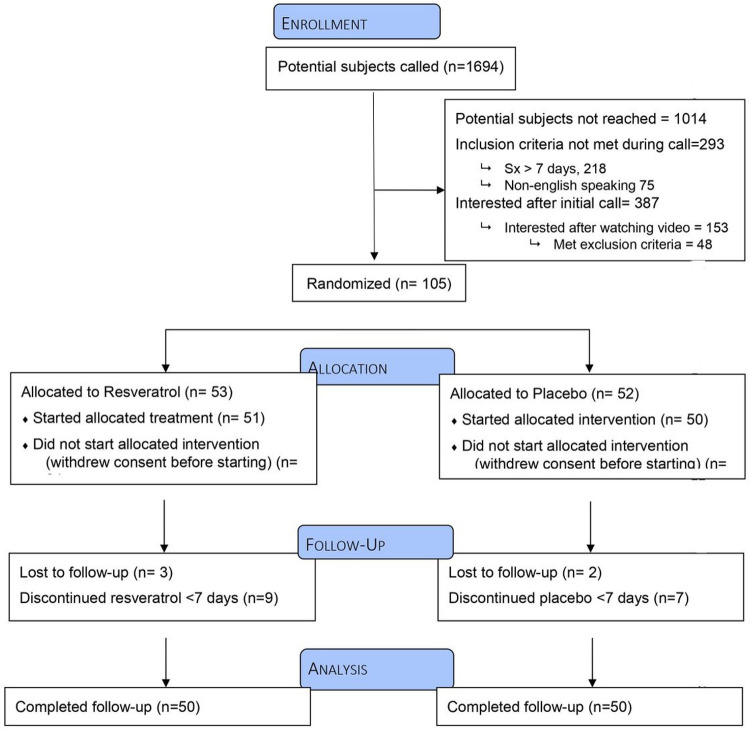
CONSORT Diagram

Between September 13, 2020 and December 11, 2020, 1,694 patients were telephoned within 24 hours of testing positive for COVID-19 to be recruited into the clinical trial (Figure 2). One-hundred-five were enrolled and randomized (Table 1). Five withdrew after receiving treatment packets (four withdrew before starting treatment and one withdrew after one treatment day citing “too many pills” as reason for withdrawal).

There was no indication of systematic biases in randomization: 4 / 122 = 3% of hypothesis tests comparing baseline symptoms between randomized groups were statistically significant at the 5% level without adjustment for multiplicity, and none were statistically significant following a Bonferroni correction. (Supplemental Tables S-1,S-2a, and S-3a).

### Compliance

Most participants completed the course of treatment. At the exit interview, 43 of 50 (86.0%) persons in the placebo group and 41 of 50 (82.0%) in the resveratrol group reported having completed at least 7 days of their respective treatments (Fisher’s exact test p-value = 0.786).

### Primary endpoint - hospitalization within 21 days

One patient (2%) in the RV group and 3 (6%) in the placebo group were hospitalized within 21 days of symptom onset (risk ratio (RR) = 0.33; 95% confidence interval (CI) = 0.04-3.10; Risk difference = −4.0%; 95% CI: (−11.6% - 3.6%); Fisher’s exact test p-value = 0.617; see Table 2). Tipping point analysis of missing outcome data indicate that no possible combination of outcomes among the five patients whose data are missing would have yielded a p-value below 0.05. Imputing outcomes that would show the strongest case for RV efficacy (both placebo patients with unobserved outcomes being hospitalized and none of the three RV patients with unobserved outcomes being hospitalized) would yield RR = 0.20; 95% CI: 0.02-1.62; p-value: 0.113; see Supplemental Table S-4. Supplemental Table S-5 shows outcomes stratified by patient characteristics. The differences in rates between study groups are not significant in any subgroup.

### Secondary endpoints

Among secondary endpoints, there were fewer events in the RV group than the placebo group for incidence of pneumonia and for emergency room visits due to COVID (Table 2). Neither difference was statistically significant. There was one pulmonary embolism in each group, so those incidence rates were equal across study groups. There were no events and therefore no differences between study groups, for death, invasive ventilation, or ICU admission. If outcomes had been observed for the five patients who withdrew from the study, no secondary endpoint could have had a statistically significant difference between study groups, even if the five outcomes had been as favorable as possible for RV (Supplemental Table S-4).

### Notable events

One patient in the placebo group was diagnosed with pancreatitis that was attributed to COVID-19 by the patient's emergency department physician.

### Adverse events

There were no serious adverse events reported. There were no significant differences in the proportion of patients from each study group reporting symptoms in a daily diary (Supplemental Table S-2b). When asked to think back over the previous seven days, the placebo group reported more severe dry mouth and more frequent general pain than the RV group, and the latter reported more frequent diarrhea (87.2% vs. 61.3%; p=0.040) and more frequent nausea (23.1% vs. 5.7%; p=0.050) than patients in the control group (Supplemental Table S-3b). No adjustments were made for multiple comparisons and only four p-values were statistically significant out of 110 symptom comparisons for study days 2-21.

## Discussion

Resveratrol is an extensively studied plant phytoalexin that has demonstrated potential beneficial biologic effects in multiple human clinical trials. With respect to COVID-19, multiple publications have suggested its use in humans as a potential treatment. This has been supported by prior research describing resveratrol’s poly-mechanistic properties; computerized molecular docking analysis demonstrating resveratrol potential to interfere with coronavirus; as well as multiple *in vitro* studies demonstrating efficacy against MERS-CoV and SARS-CoV-2.

It should be noted that the much of the resveratrol literature is concerned about poor bioavailability and discounts possible effects of resveratrol metabolites such as the more intravascularly abundant resveratrol-glucuronides ^60,61^. This dismissal of resveratrol’s metabolites is despite the fact that other drugs have demonstrated increased potency in their metabolized forms (i.e., morphine-6-glucuronide is known to be more potent than morphine) ^62^. Molecular docking analysis suggest that resveratrol-glucuronide may be more potent against coronavirus since there is a higher binding affinity between resveratrol-glucuronides and coronavirus structures ^6^.

Although the primary outcome results are not statistically significant, in this phase 2, double-blind, placebo-controlled, randomized clinical trial, resveratrol was associated with a lower incidence of pneumonia, COVID-related ER visits, and hospitalization. The favorable risk ratios could be due to chance, but there are biological reasons to believe that RV would be effective and so the protective effect may be quite real, but not significant due to small sample size and low incidence of the outcomes. It is notable that in influenza, shorter time between the onset of symptoms and the start of antiviral treatment results in improved outcomes such that they CDC primarily recommends starting treatments within 48 hours ^63^. The median time from symptom onset to delivery of treatment packet was 5 days. The magnitude of effect of resveratrol in COVID might be greater if treatment could be started sooner, but due to delays in presentation, test results, and delivery, a 48-hour treatment window was not feasible for this study. There were no serious adverse events attributed to resveratrol in this study, and given resveratrol’s long safety history, the data presented here would support a larger clinical trial to determine efficacy, ideally starting treatment shortly after the onset of symptoms.

While the results of this study were largely underpowered due to small sample size, there were a few measures that did reach statistical significance. Dry mouth (p=0.046), nausea (p=0.05), and diarrhea (p=0.04) was reported in higher frequency in the RV group. This is certainly consistent with known gastrointestinal side effects of resveratrol.

Resveratrol treated patients had a lower incidence of overall pain (p=0.04). This is consistent with prior preclinical literature demonstrating RV to have analgesic properties as a cyclooxygenase inhibitor (COX I & COX II) ^64^. This would also support that orally administer resveratrol is able to achieve system effects despite concerns for limited bioavailability.

This study was a proof-of-concept to primarily determine the safety of using resveratrol in the setting of COVID-19, noting the FDA guidance was to limit this study to no more than 200 participants with a planned interim safety analysis after the first 100 patients were enrolled. Enrollment in the study was slow initially but did rapidly increase in December as Ohio was starting its third COVID-19 wave. Enrollment was paused after the 100^th^ patient so that an interim analysis could be performed. After completion of data collection and an interim analysis by an independent Data Safety Monitoring Board, Ohio’s third COVID-19 wave was ending. While the DSMB did approve continuation of the study, a feasibility analysis of daily case rate in the Mount Carmel Health System, and considering the prior rate of enrollment, it was estimated that it would take at least another 6-8 months to enroll another 100 patients. The enrollment rate would further be impacted by the availability of vaccinations and competing treatments (such a monoclonal antibodies). Furthermore, a statistical futility analysis also suggested that 100 more patients would be inadequate to determine efficacy, therefore the study was discontinued after the first 100 patients.

Additional limitations include limited geographic area, limited racial diversity, and a disproportionate number of heath care providers as subjects in the trial.

While 100 percent of the participants were contacted to determine their primary outcome measures, compliance with PRO-CTCAE was limited, resulting an incomplete picture of adverse events. A better funded clinical trial with larger research staff might be more effective at achieving patient compliance through more active patient contact (such as routine phone calls throughout the study).

## Conclusion

This proof-of-concept study, along with the wealth of other resveratrol pre-clinical research, supports further investigation resveratrol as a potential treatment of COVID-19 and possibly other viral respiratory infections (including influenza, Respiratory Syncytial Virus, and Human Rhinovirus) ^65^. If the magnitude of the effect of this small study was representative of a larger trial, the number needed to treat to prevent ER visits or hospitalization would compare favorably against currently available (i.e., monoclonal antibody therapy) outpatient treatments.

Given the scale of the health and economic impacts of COVID-19, any treatment that can reduce hospitalizations could have a significant impact in this pandemic. RV is generally recognized as safe and has been shown to have positive health benefits in human trials. Prior research in human trials related to lung disease, in vitro studies of RV of coronavirus, and animal studies of RV in other viral infections support investigating RV as a treatment for coronavirus disease. Given that RV is readily available and could be cheaply scaled up through the fermentation of yeast, it is potentially a scalable solution to treat COVID-19.

## Supplementary Material

Supplement 1

## Figures and Tables

**Table 1:** Characteristics of participants.

	Placebo (N=52)	Resveratrol (N=53)	Overall (N=105)
Age			
Mean (SD)	55.7 (8.55)	56.3 (9.46)	56.0 (8.98)
Median [Min, Max]	54.0 [45.0, 75.0]	55.0 [45.0, 84.0]	55.0 [45.0, 84.0]
Sex			
Male	19 (36.5%)	24 (45.3%)	43 (41.0%)
Female	33 (63.5%)	29 (54.7%)	62 (59.0%)
Race			
White	46 (88.5%)	47 (88.7%)	93 (88.6%)
Black	2 (3.8%)	2 (3.8%)	4 (3.8%)
Multiple	3 (5.8%)	1 (1.9%)	4 (3.8%)
Asian	0 (0%)	1 (1.9%)	1 (1.0%)
Other	0 (0%)	1 (1.9%)	1 (1.0%)
Did not answer	1 (1.9%)	1 (1.9%)	2 (1.9%)
Ethnicity			
Hispanic/Latino	1 (1.9%)	1 (1.9%)	2 (1.9%)
Not Hispanic/Latino	45 (86.5%)	43 (81.1%)	88 (83.8%)
Not specified	6 (11.5%)	9 (17.0%)	15 (14.3%)
BMI			
Mean (SD)	31.4 (7.32)	29.1 (4.68)	30.2 (6.20)
Median [Min, Max]	30.0 [21.6, 58.9]	28.5 [19.8, 42.7]	29.3 [19.8, 58.9]
Missing	1 (1.9%)	1 (1.9%)	2 (1.9%)
High-risk comorbidity			
Yes	17 (32.7%)	15 (28.3%)	32 (30.5%)
No	35 (67.3%)	38 (71.7%)	73 (69.5%)
ACE inhibitor / ACE receptor blocker			
Yes	10 (19.2%)	5 (9.4%)	15 (14.3%)
No	42 (80.8%)	48 (90.6%)	90 (85.7%)

SD = standard deviation; ACE = Angiotensin-converting enzyme

**Table 2:** Primary and secondary outcomes, as observed, by study group.

	Placebo	Resveratrol	Risk Ratio Risk Difference	95% CI	p-value
	N (%)	N (%)			
**Primary outcome**					
**Hospitalization**	3 (6.0)	1 (2.0)	0.33 −4.0%	0.04-3.10 −11.6-3.6%	0.617
**Secondary outcomes**					
**Death**	0 (0)	0 (0)	NA	NA	1
**Invasive ventilation**	0 (0)	0 (0)	NA	NA	1
**ICU admission**	0 (0)	0 (0)	NA	NA	1
**ER visits for COVID**	7 (14.0)	4 (8.0)	0.57 −6.0%	0.18-1.83 −18.2-6.2%	0.525
**Pneumonia**	8 (16.0)	4 (8.0)	0.50 −8.0%	0.16-1.55 −20.6-4.6%	0.357
**Pulmonary embolism**	1 (2.0)	1 (2.0)	1.00 0%	0.06-15.55 −5.5-5.5%	1

All outcomes evaluated over the 21 days that followed patient randomization to study group.

Outcomes observed for N=50 patients per group.

NA = not applicable; CI = confidence interval; ICU = intensive care unit; ER = emergency room

P-value from Fisher's exact test.

## Data Availability

Deidentified individual data that supports the results will be shared by written request to the communicating author; provided the requesting investigator has approval from an Institutional Review Board (IRB), Independent Ethics Committee (IEC), or Research Ethics Board (REB), as applicable, and executes a data use/sharing agreement with Mount Carmel Health System.
